# Functional MRI Preprocessing in Lesioned Brains: Manual Versus Automated Region of Interest Analysis

**DOI:** 10.3389/fneur.2015.00196

**Published:** 2015-09-25

**Authors:** Kathleen A. Garrison, Corianne Rogalsky, Tong Sheng, Brent Liu, Hanna Damasio, Carolee J. Winstein, Lisa S. Aziz-Zadeh

**Affiliations:** ^1^Department of Psychiatry, Yale School of Medicine, New Haven, CT, USA; ^2^Division of Biokinesiology and Physical Therapy, University of Southern California, Los Angeles, CA, USA; ^3^Brain and Creativity Institute, University of Southern California, Los Angeles, CA, USA; ^4^Department of Speech and Hearing Science, Arizona State University, Tempe, AZ, USA; ^5^Palo Alto VA Medical Center, Palo Alto, CA, USA; ^6^Stanford University School of Medicine, Palo Alto, CA, USA; ^7^Department of Biomedical Engineering, University of Southern California, Los Angeles, CA, USA; ^8^Department of Psychology, University of Southern California, Los Angeles, CA, USA; ^9^Division of Occupational Science and Occupational Therapy, University of Southern California, Los Angeles, CA, USA

**Keywords:** stroke, lesion, spatial normalization, inferior frontal gyrus, region of interest analysis

## Abstract

Functional magnetic resonance imaging (fMRI) has significant potential in the study and treatment of neurological disorders and stroke. Region of interest (ROI) analysis in such studies allows for testing of strong *a priori* clinical hypotheses with improved statistical power. A commonly used automated approach to ROI analysis is to spatially normalize each participant’s structural brain image to a template brain image and define ROIs using an atlas. However, in studies of individuals with structural brain lesions, such as stroke, the gold standard approach may be to manually hand-draw ROIs on each participant’s non-normalized structural brain image. Automated approaches to ROI analysis are faster and more standardized, yet are susceptible to preprocessing error (e.g., normalization error) that can be greater in lesioned brains. The manual approach to ROI analysis has high demand for time and expertise, but may provide a more accurate estimate of brain response. In this study, commonly used automated and manual approaches to ROI analysis were directly compared by reanalyzing data from a previously published hypothesis-driven cognitive fMRI study, involving individuals with stroke. The ROI evaluated is the pars opercularis of the inferior frontal gyrus. Significant differences were identified in task-related effect size and percent-activated voxels in this ROI between the automated and manual approaches to ROI analysis. Task interactions, however, were consistent across ROI analysis approaches. These findings support the use of automated approaches to ROI analysis in studies of lesioned brains, provided they employ a task interaction design.

## Introduction

Functional magnetic resonance imaging (fMRI) is used often in neuroscience and clinical practice (1) to study human brain function both in clinical and healthy populations. Studies in individuals with stroke, for example, use fMRI to evaluate changes in brain function and to relate these to changes in behavior, to estimate clinical outcomes and to attempt to reduce disability [e.g., Ref. (2, 3)]. fMRI is also used to evaluate therapeutic efficacy, for example, of stroke neurorehabilitation approaches [e.g., Ref. (4)], or to provide proof-of-concept for novel clinical therapeutics (5, 6). fMRI has the potential to provide important information about individualized treatments and outcomes (1).

A major challenge to the use of fMRI in clinical research and clinical practice is that it can be difficult to obtain robust enough signals in individuals to allow confident evaluations (1). In whole brain analyses, blood oxygen level-dependent (BOLD) signal is measured in each voxel and fit to a general linear model (GLM), and the test statistics are computed across thousands of voxels, leading to the well-known problem of multiple comparisons (7). One way to improve statistical power is to limit the number of tests; in neuroimaging, this can be accomplished by restricting analysis to an *a priori* ROI (8). In addition, corrections for multiple comparisons used to control for false positives resulting from correlation across voxels (e.g., Bonferroni correction) can be applied to an ROI or a small number of ROIs rather than to the thousands of voxels in the whole brain (8). Strong prior hypotheses are required in order to take advantage of the ROI approach. In clinical practice, ROI analyses can be useful when intervention decisions are linked to strong prior hypotheses about individual brain regions (9).

One approach for ROI analysis is to identify regions based on anatomical landmarks and define ROIs on each individual’s structural brain image (10–14). This manual approach accommodates the inter-subject variability in neuroanatomy (8), including variance related to brain lesions, and may be considered the gold standard for studies in clinical populations. However, manual definition of ROIs is highly time consuming, labor intensive, and has to rely on expertise; therefore, it has the risk of inter-experimenter variability. In addition, while some brain regions may be defined reliably, others lack clear anatomical landmarks and therefore may be more difficult to demarcate.

An alternative approach is to identify ROIs based on automatic demarcation using anatomical atlases or template brains. To do so, each individual’s structural brain image must be first registered to the standard space defined by the template brain image; such registration is achieved by spatial normalization (15). In clinical populations, however, structural changes, such as brain lesions or loss of brain volume, may result in a lack of perfect correspondence between the individual’s brain image and the template brain image, not allowing a perfect spatial normalization process (16). Such suboptimal normalization may lead to misalignment and therefore decreased sensitivity within a group of subjects, or worse, to false attribution of group differences to brain functional rather than structural differences (16, 17). Normalization errors are always greater in damaged brains, introducing a confound in comparisons between patients and control groups. Spatial normalization algorithms attempt to reduce image mismatches, and the solutions have been improving. For example, a recent approach uses unified segmentation and normalization (18) to estimate the model parameters used to fit an individual’s brain to standard space by alternating between image bias correction, tissue segmentation, and non-linear registration; an approach that has been optimized for lesioned brains (16). Nevertheless, differences between automated and manual ROI analysis have not been well quantified, especially in brains with structural lesions.

This study examined the results of a new ROI analysis of a previously published hypothesis-driven cognitive fMRI study involving individuals with stroke (5), comparing commonly used approaches to automated and manual ROI analysis. The cognitive task employed in that study was action observation; therefore, the ROI examined in the current study was the inferior frontal gyrus pars opercularis (Brodmann area, BA 44, the posterior half of Broca’s area), considered to be part of the putative human mirror neuron system that is activated during both action execution and action observation (19, 20). This brain region is also implicated in a wide range of functions, including speech production (21) and perception (22, 23), and working memory (24, 25), among others. This ROI has potential clinical significance in utilizing action observation in stroke rehabilitation (26, 27), measuring reactivation of language networks during stroke recovery (28, 29), and as a focus of pre-surgical language network mapping (30). The current study compared activity in BA 44 using an automated and manual approach to ROI analysis. In the automated method, individual brain images were spatially normalized to a template brain image and ROIs were automatically defined in the standard space. In the manual method, ROIs were hand-drawn on each individual’s non-normalized structural brain image using neuroanatomical landmarks. Differences in task-related effect size and percent-activated voxels between the automated and manual methods were characterized and compared between lesioned and control brains.

## Materials and Methods

### Participants

Twelve individuals with stroke (six females; mean age 66 years, range 40–86 years) and 12 control individuals (five females, mean age 66 years, range 40–82 years) participated in the prior study (5). All participants were right-handed (31) and had normal or corrected vision. All participants with stroke had chronic strokes (mean duration 8 years, range 2–17 years) of the middle cerebral artery in the dominant left hemisphere. Six individuals had lesions involving the left internal capsule, and six individuals had lesions involving the left frontal cortex and internal capsule. Automated Lesion Identification (32) was used to estimate lesions and visualize lesion overlap [For lesion overlap map, see Ref. (5)]. Four of the six individuals with frontal cortical lesions had lesions involving the ROI analyzed in this study (mean overlap between lesion and the left inferior frontal gyrus = 6% ± 4.7 (SEM); range 0.2–56.5%). All participants with stroke had moderate to severe right upper limb hemiparesis [mean Fugl-Meyer Assessment of the Upper Extremity score = 29.8 ± 4.1 (SEM); range 13–48; Ref. (33)]. Informed consent was obtained according to the Declaration of Helsinki and the institutional review board of the University of Southern California.

### Imaging procedure

Images were acquired using a 3 T Siemens Trio MRI. T1-weighted anatomical images were acquired for participants with stroke (TR = 2350 ms, TE = 3.09 ms, 256 mm × 256 mm, 208 slices, slice thickness 1 mm, flip angle = 10°, 1 mm × 1 mm × 1 mm) and for healthy participants (TR = 1950 ms, TE = 2.26 ms, 256 mm × 256 mm, 176 slices, slice thickness 1 mm, flip angle = 9°, 1 mm × 1 mm × 1 mm). All participants took part in a hypothesis-driven fMRI study of action observation (5). Functional MRI included four 12 s blocked conditions: (a) right hand action observation, (b) left hand action observation, (c) static images of hands, and (d) rest. During action observation, participants watched videos of an actor grasping objects using either his right hand or his left hand. Participants were instructed to remain still and pay attention to the videos. Prior to actual scanning, the procedure was practiced in a mock scanner. During scanning, all participants were visually monitored for movement; no overt movement was detected in any participant. Each condition block was repeated 15 times followed by rest, randomized across three 6 min runs (gradient echo, TR = 2 s, TE = 30 ms, 64 mm × 64 mm, 37 slices, flip angle = 90°, 3.5 mm × 3.5 mm × 3.5 mm).

### Imaging analysis

#### Image Preprocessing and Analysis

Image preprocessing was conducted using SPM12.^1^ Functional images were realigned for motion correction and the resultant motion parameters were included as regressors of no interest in the fMRI model. In addition, Artifact detection tools (ART)^2^ was used to identify mean global intensity and motion outliers in the fMRI time series using an outlier threshold of global signal >3 SDs and motion >1 mm, and the detected outliers were included as regressors of no interest in the fMRI model. For the manual ROI analysis, functional images were smoothed using a 6 mm full width half maximum (FWHM) Gaussian kernel, and no further preprocessing steps were taken. For the automated ROI analysis, the structural image was segmented and all images were normalized using SPM12’s unified segmentation normalization (18), and smoothed using a 6 mm FWHM Gaussian kernel. Both approaches utilized smoothing in order to improve signal to noise (34). First level models were specified to estimate the betas for each participant for the conditions of right hand and left hand action observation separately. Rest was modeled as implicit baseline.

#### Region of Interest Definition

**Figure 1:**
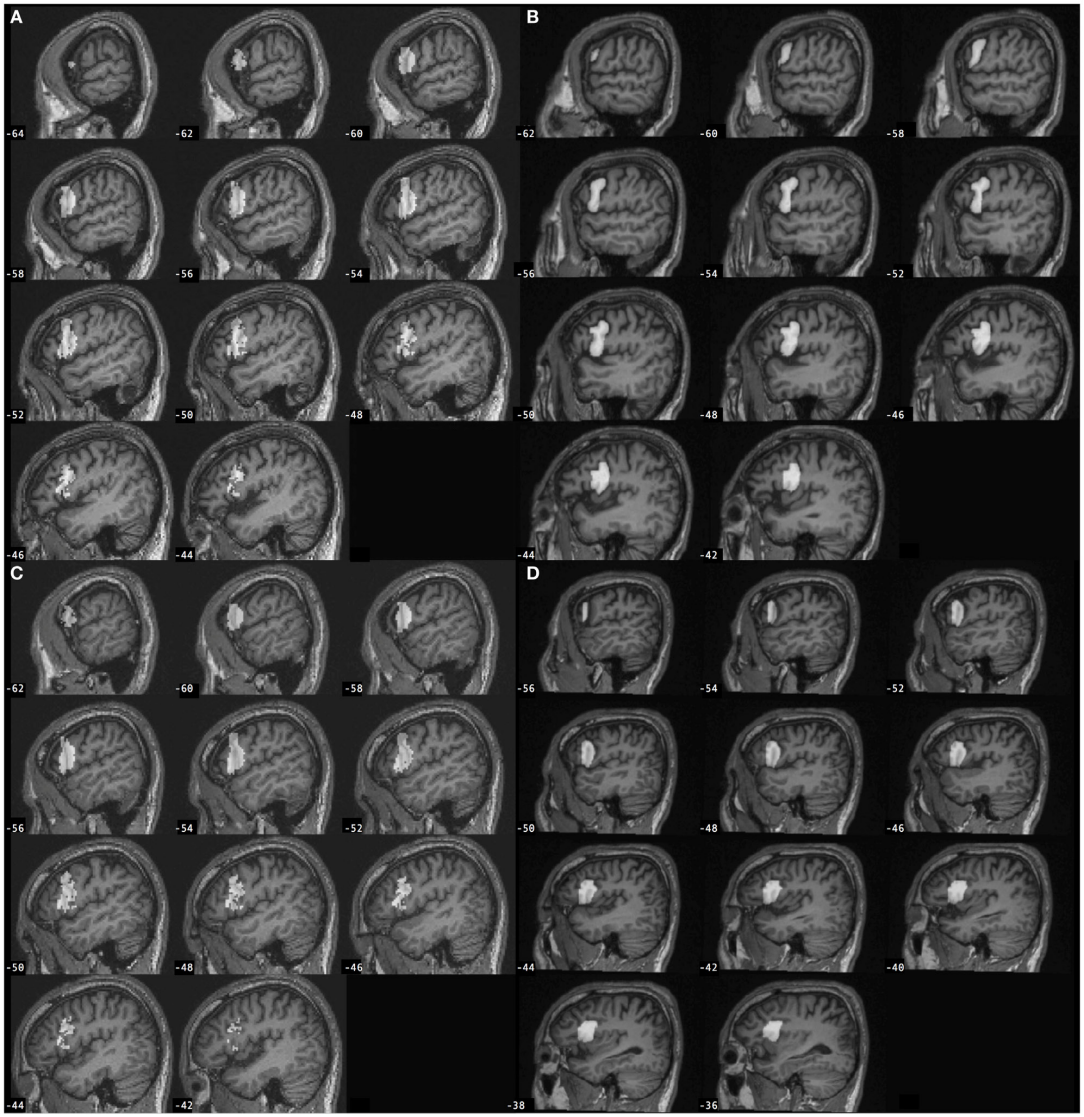
**Left inferior frontal gyrus pars opercularis (BA 44) regions of interest (gray) defined for a participant with stroke according to the (A) automated approach and (B) manual approach, and for a control participant according to the (C) automated approach and (D) manual approach**. Automated BA 44 was defined from SPM Anatomy toolbox and is overlaid onto the individual participant’s spatially normalized brain image. Manual BA 44 was defined by-hand based on neuroanatomy and is overlaid onto the individual participant’s non-normalized brain image.

Region of interest (ROIs) included the left and right pars opercularis of the inferior frontal gyrus (BA 44). The methods to define ROIs using the manual approach and the automated approach are described below. Examples of ROIs defined using each of the approaches are displayed in Figure 1.

##### Manual region of interest definition

Region of interest were defined manually on each individual’s non-normalized structural brain image based on neuroanatomical landmarks using the principles described by Allen et al. (35, 36). The following landmarks were used to define the pars opercularis of the inferior frontal gyrus (BA 44): ascending branch of the Sylvian fissure (anterior boundary), inferior frontal sulcus (dorsal boundary), precentral sulcus (posterior boundary), Sylvian fissure (ventral boundary), depth of the ascending branch of the Sylvian fissure and depth of the inferior frontal sulcus (medial boundary). ROIs were hand-drawn using MRIcron^3^ (37) by a research assistant with no further involvement in the study, who had been trained to identify and trace neuroanatomical landmarks and lesion boundaries due to stroke, and who was blinded to the purpose of the current study, i.e., did not know that the manual drawings would be compared to an automated approach. ROI drawings were checked by an investigator (KAG); reviewed by a researcher with extensive neuroanatomy experience including related to BA 44 [CR; (22, 23)]; overseen by an expert MRI neuroanatomist [HD; (38)]; and adjusted where necessary in discussion with the research team.

##### Automated region of interest definition

Automated ROI definition used the left and right BA 44 defined regions in SPM Anatomy toolbox (39) defined in Montreal Neurological Institute (MNI) space (40). In SPM Anatomy toolbox, anatomical regions have been defined based on maximum probability cytoarchitectonic maps.

##### Region of interest volume and overlap

Region of interest volume was calculated using fslstats from Fslutils (41).^4^ ROI overlap was evaluated by first reslicing the ROIs into the same MNI space using SPM12. Spatial agreement between each manual ROI and the automated ROI for the same hemisphere was then calculated using Dice’s coefficient (42), a measure of the volume of the overlap relative to the mean volume of the two ROIs, using a Matlab script (provided by Chris Rorden).^5^ Dice’s coefficient (*d*) ranges from 0 (no overlap) to 1 (complete overlap). ROI overlap was visualized using fslmaths and MRIcron.

#### Region of Interest Analysis

Region of interest were analyzed using MarsBar (43) to (1) extract all of the data within the ROI for each functional image to provide a voxel time course for each voxel in the ROI, (2) calculate a summary time course for each ROI as the mean of all voxel values in the ROI, (3) estimate the fMRI model with the ROI data according to SPM12’s implementation of the GLM, (4) apply a contrast (e.g., “task minus rest”) to the estimated model to derive an effect size for each contrast, and (5) extract the percent of activated voxels in the ROI (*T* = 1.7–5.0). Contrasts tested in this study included the main effects of right hand and left hand action observation, separately.

### Statistical analysis

One-sample *t*-tests were used to test whether the volume of ROIs defined manually differed from the volume of the automated ROI for each hemisphere, for each group. Repeated measures analyses of variance (ANOVA) were used to determine a difference in effect size or percent of activated voxels between automated and manual approaches to ROI analysis, with method (automated/manual), condition (right hand/left hand action observation), and hemisphere (right/left) as within-subject factors, and group (stroke/control) as a between-subjects factor. Paired *t*-tests were used *post hoc* to determine differences between automated and manual approaches to ROI analysis for each group, hemisphere, and condition, for descriptive purposes.

## Results

### Spatial overlap between manual and automated ROI definition

**Figure 2:**
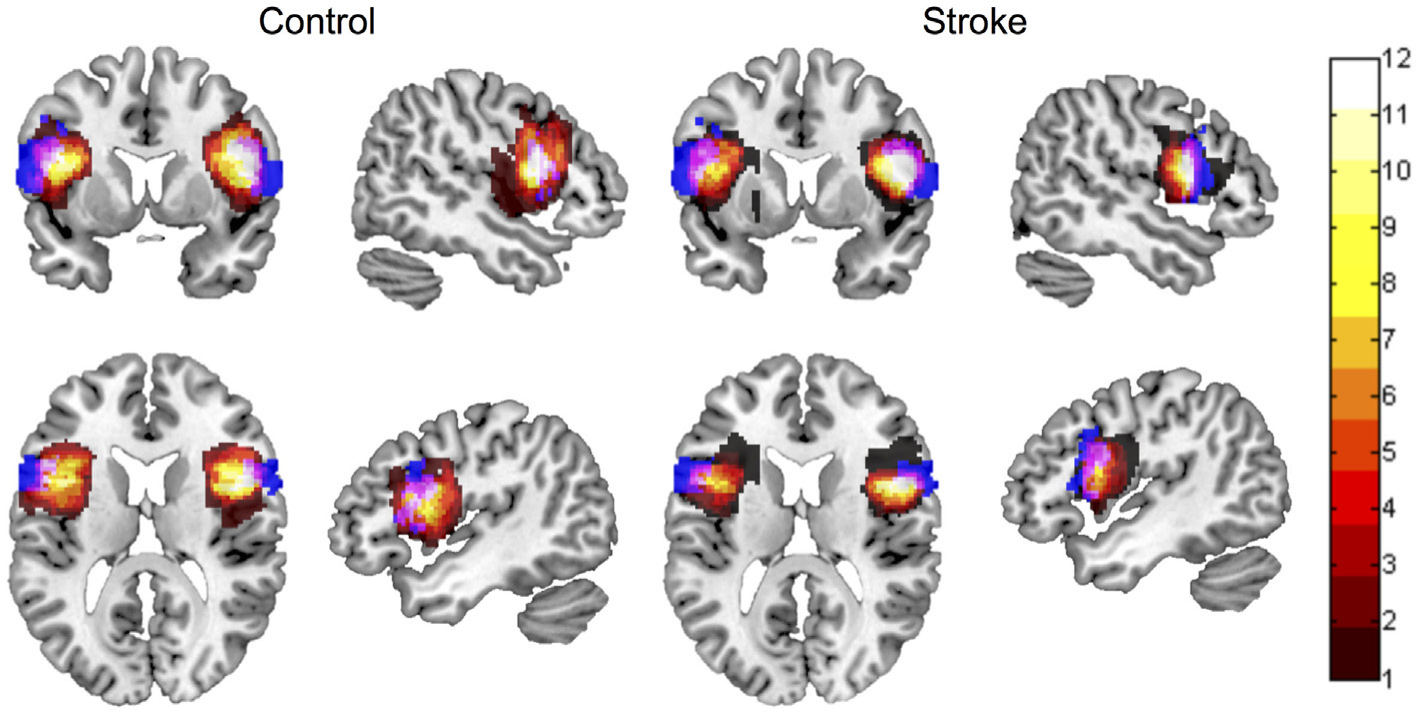
**Spatial overlap between ROI maps for the left and right inferior frontal gyrus pars opercularis (BA 44), for control participants and participants with stroke**. ROI maps defined manually are displayed in hot (color bar indicates 1–12 participants in each group). ROI maps defined using the automated approach are displayed in blue. Spatial overlap between manual and automated maps is indicated in pink. ROIs are overlaid onto the MNI template brain image in neurological orientation.

Spatial overlap between manually defined ROIs and the automated ROI for left BA 44, as evaluated using Dice’s coefficient, was *d* = 0.2 ± 0.1 in lesioned brains, and *d* = 0.16 ± 0.07 in control brains. Spatial overlap between manually defined ROIs and the automated ROI for right BA 44 was *d* = 0.21 ± 0.1 in lesioned brains, and *d* = 0.17 ± 0.1 in control brains. Spatial overlap between manual and automated ROIs is displayed in Figure 2.

### Volume differs between manual and automated ROI definition

ROI volume for the left BA 44 was significantly smaller when defined manually compared to the automated ROI (automated = 9326 mm^3^), both in lesioned brains (manual = 6420 (mean) ± 614 mm^3^ (SEM); *t* = −4.73, *p* = 0.001) and in control brains (manual = 6267 ± 691 mm^3^; *t* = −4.43, *p* = 0.001). ROI volume for the right BA 44 was not significantly different when defined manually compared to the automated ROI (automated = 7012 mm^3^), neither in lesioned brains (manual = 6102 ± 492 mm^3^; *t* = −1.85, *p* = 0.091) nor in control brains (manual = 7963 ± 720 mm^3^; *t* = 1.32, *p* = 0.214).

### Effect size differs between manual and automated approaches to ROI analysis

**Figure 3:**
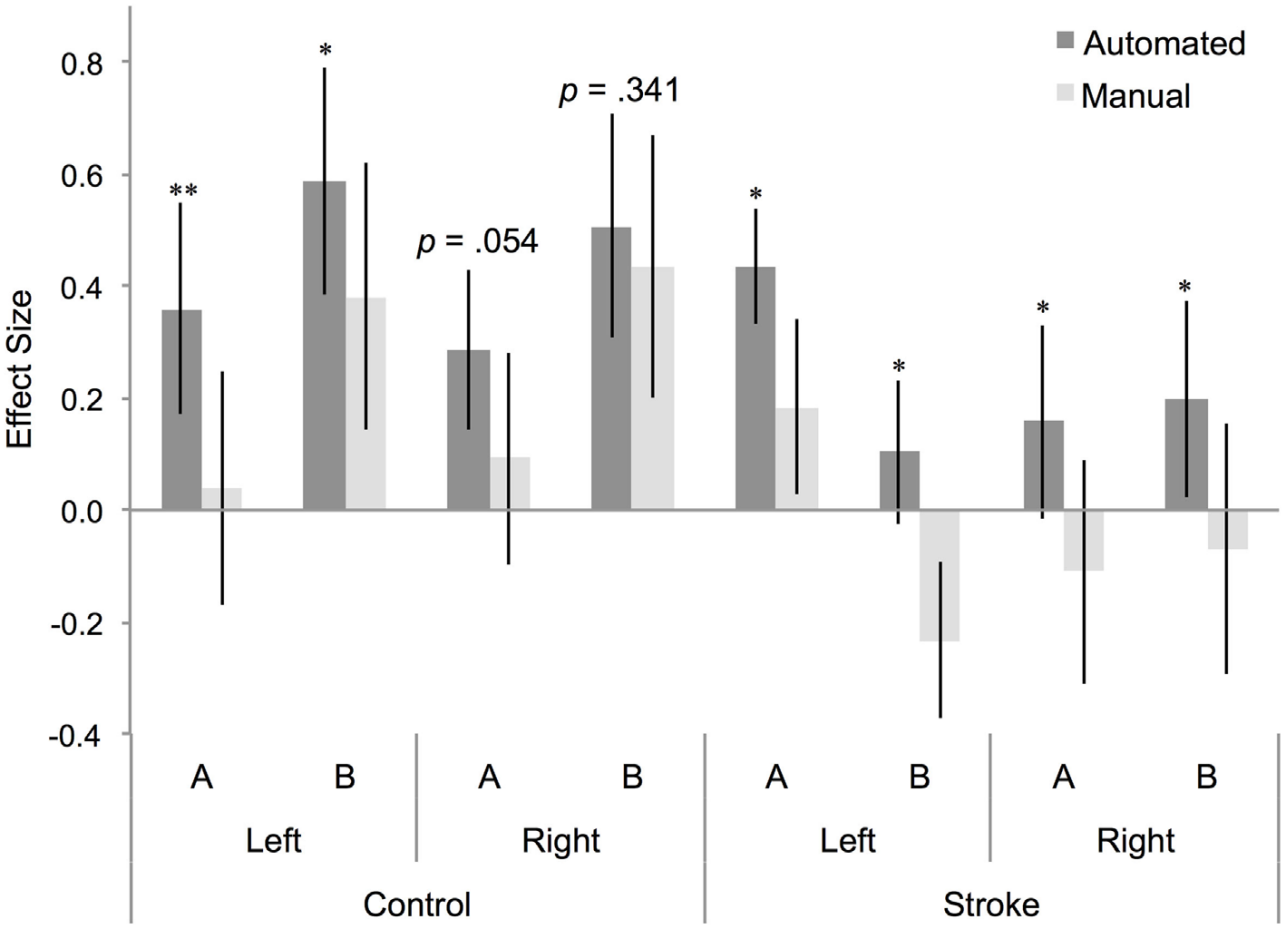
**Effect size for BA 44 differs between manual and automated approaches to ROI analysis**. Effect size for control participants and participants with stroke, for BA 44 in the left and right hemisphere, for (A) right hand action observation and (B) left hand action observation, using the automated approach to ROI analysis (dark gray bars) and the manual approach to ROI analysis (light gray bars). Error bars indicate SEM. *p < 0.05, **p < 0.01.

**Figure 4:**
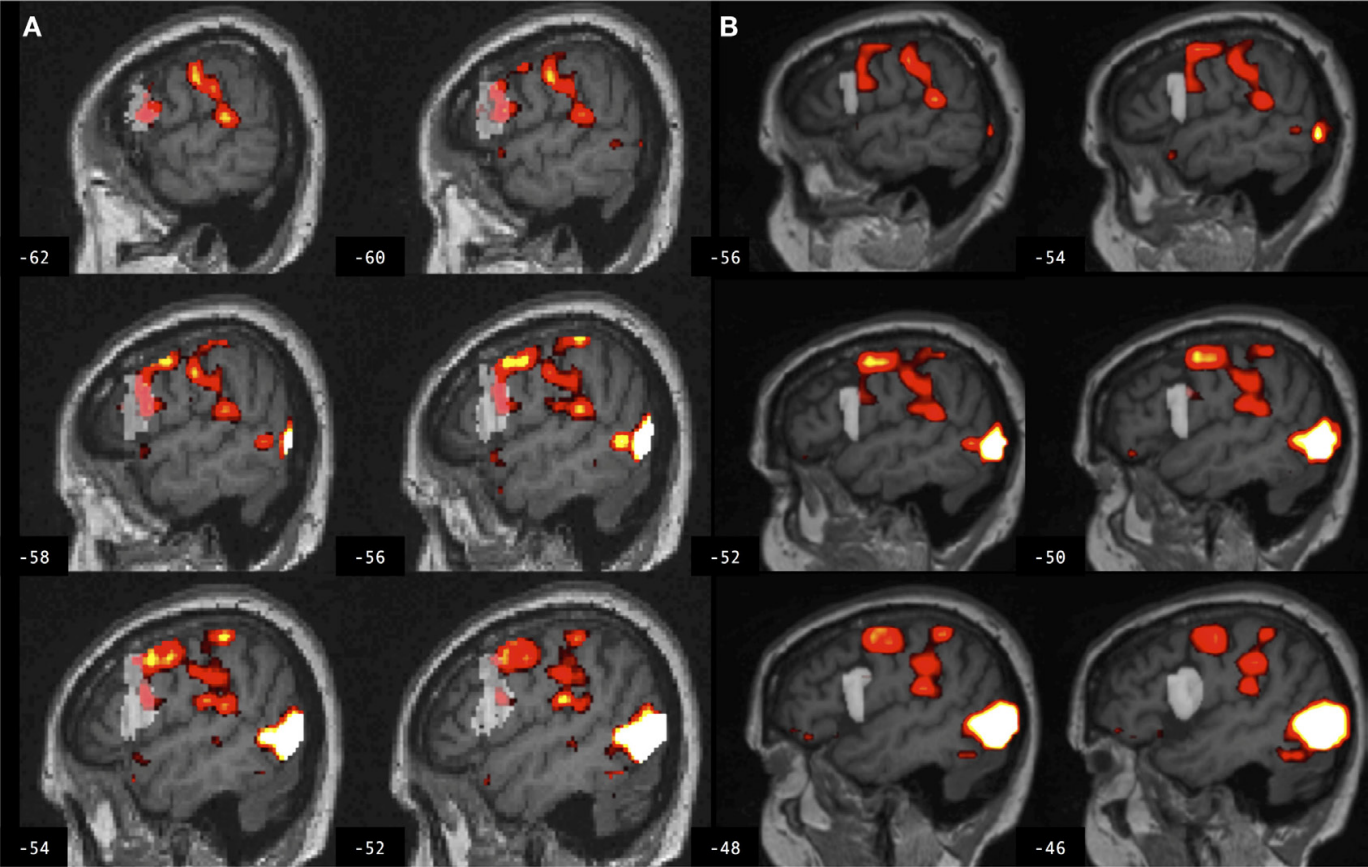
**Brain activation associated with right hand action observation in the left hemisphere of a control participant as evaluated by: (A) a commonly used automated approach to ROI analysis, normalized and overlaid onto the MNI brain image; and (B) a manual approach to ROI analysis, overlaid onto the participant’s non-normalized brain image**. ROI masks for the left BA 44 are displayed in gray. In this example, the larger automated ROI in **(A)** captured a larger number of activated voxels than the smaller manually defined ROI in **(B)**. For display, activation maps are shown at *T* = 1.67–10 corresponding to *p* < 0.05 uncorrected.

**Figure 5:**
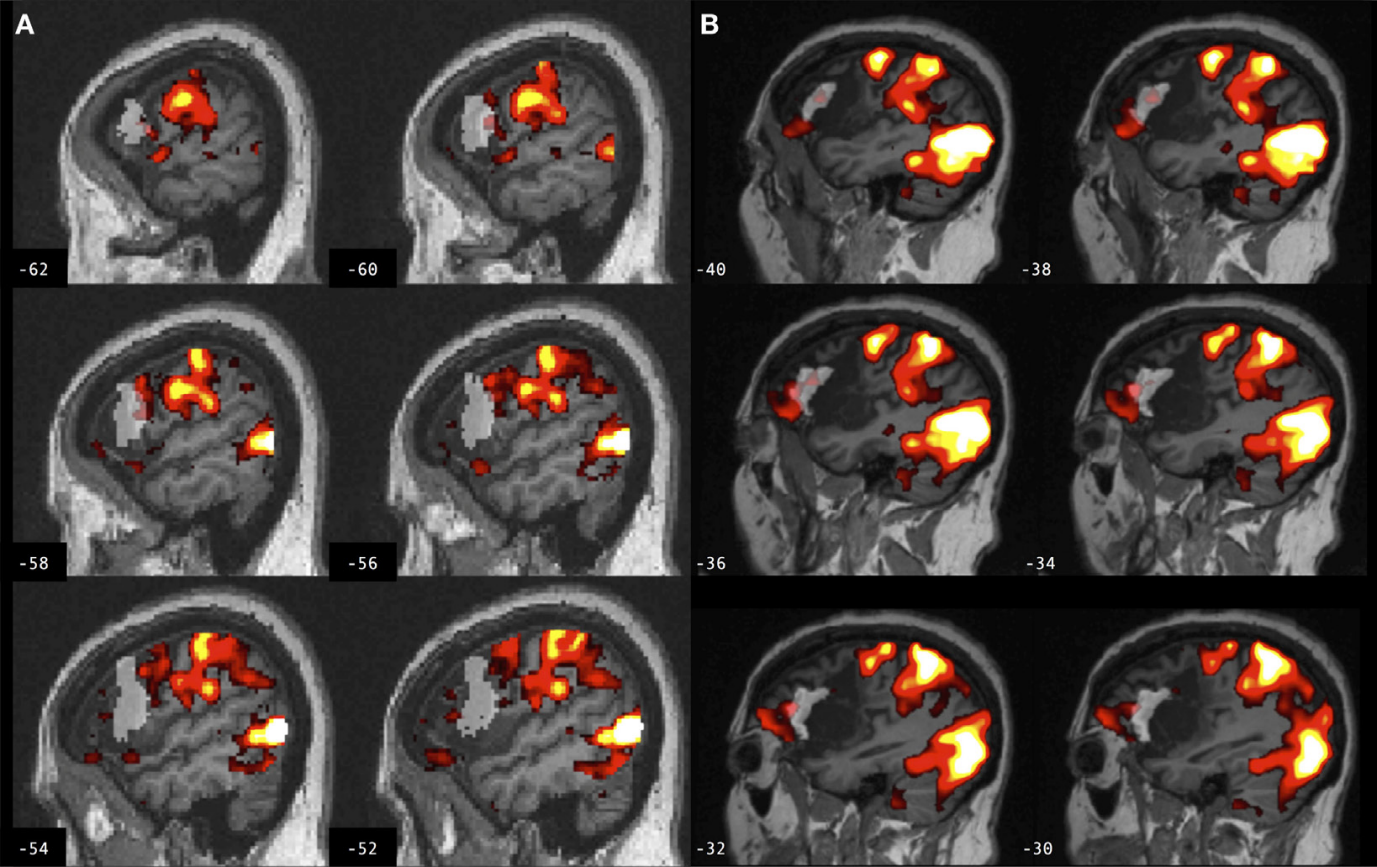
**Brain activation associated with right hand action observation in the left hemisphere of a participant with stroke involving the cortex and internal capsule, as evaluated by: (A) a commonly used automated approach to ROI analysis, normalized and overlaid onto the MNI brain image; and (B) a manual approach to ROI analysis, overlaid onto the participant’s non-normalized brain image**. ROI masks for left BA 44 are displayed in gray. In this example, the automated ROI in **(A)** does not contain the intact tissue from left BA 44, whereas the experimenter was able to demarcate the displaced tissue in the manually defined ROI in **(B)**. For display, activation maps are shown at *T* = 1.67–10 corresponding to *p* < 0.05 uncorrected.

**Figure 6:**
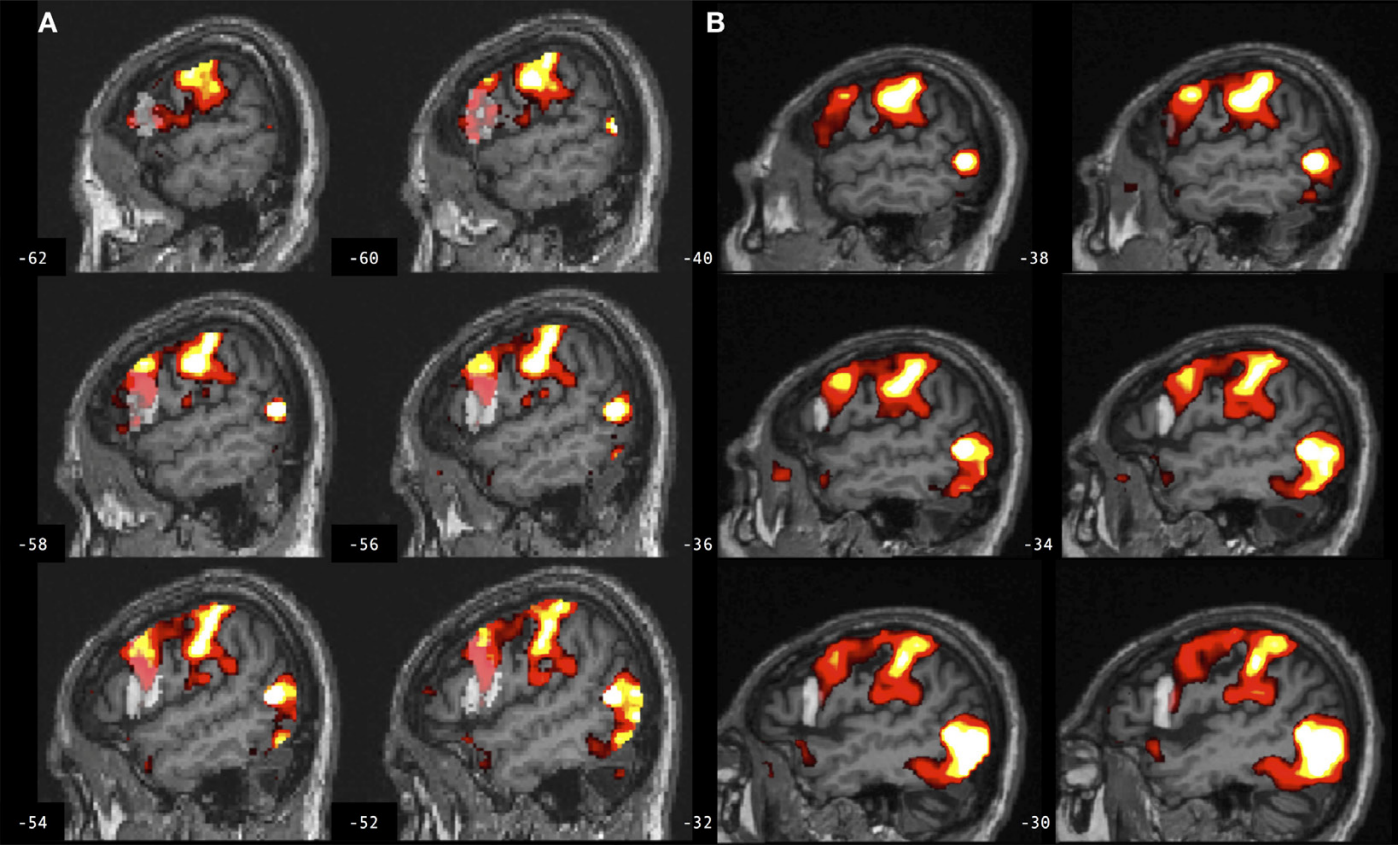
**Brain activation associated with right hand action observation in the left hemisphere of a participant with stroke involving the internal capsule, as evaluated by: (A) a commonly used automated approach to ROI analysis, normalized and overlaid onto the MNI brain image; and (B) a manual approach to ROI analysis, overlaid onto the participant’s non-normalized brain image**. ROI masks for left BA 44 are displayed in gray. In this example, peak activation is localized to the left ventral premotor cortex according to the manual approach in **(B)**, whereas due in part to larger ROI volume and spatial smoothing, the activation is localized to the left BA 44 according to the automated approach in **(A)**. For display, activation maps are shown at *T* = 1.67–10 corresponding to *p* < 0.05 uncorrected.

A four-way repeated measures ANOVA determined that effect size differed significantly between approaches [*F*(1,22) = 23.075, *p* = 0.000085; Table 1]. A *post hoc* pairwise comparison using the Bonferroni correction revealed a significantly larger effect size for automated as compared to manual ROI analysis (automated = 0.329 ± 0.09, manual = 0.090 ± 0.109; *p* = 0.000085; Figure 3). Pairwise comparisons for each method, group, hemisphere, and condition are displayed in Figure 3. Additional within-subjects test results are provided in Table 1. Other main effects and interactions related to the methods comparison were not significant, including interactions between method and group (*p* = 0.416), method and hemisphere (*p* = 0.231), and method, group, and hemisphere (*p* = 0.418). However, as expected based on prior analysis of this dataset (5), a significant three-way interaction was found between cognitive task condition, group, and hemisphere [*F*(1,22) = 8.438, *p* = 0.008; Table 1] that was consistent when tested as a three-way repeated measures ANOVA separately for either the automated [*F*(1,22) = 7.350, *p* = 0.013] or manual approach [*F*(1,22) = 6.824, *p* = 0.016]. Note that for participants with stroke, the left hemisphere is the lesioned hemisphere. Representative activation maps from individual participants are displayed in Figures 4–6 to illustrate the differences between automated and manual approaches to ROI definition, and are discussed in more detail below.

**Table 1:** **Effect size differs between manual and automated approaches to ROI analysis**.

Source	*F* ratio	*p*
**Method**	**23.075**	**0.000**
Method × group	0.686	0.416
Task	0.207	0.654
Task × group	3.176	0.089
Hemisphere	0.214	0.648
Hemisphere × group	0.118	0.734
Method × task	0.504	0.485
Method × task × group	2.125	0.159
Method × hemisphere	1.518	0.231
Method × hemisphere × group	0.681	0.418
**Task × hemisphere**	**8.073**	**0.010**
**Task × hemisphere × group**	**8.438**	**0.008**
Method × task × hemisphere	0.502	0.486
Method × task × hemisphere × group	0.371	0.549

*Within-subjects test results for the main effects of and interactions between methods (automated/manual), condition (right hand/left hand action observation) and hemisphere (right/left) on the effect size between groups (stroke/control). Significant effects are indicated in bold (*p* < 0.05)*.

### Percent activated voxels differs between manual and automated approaches to ROI analysis

Similarly, a four-way repeated measures ANOVA determined that percent activated voxels differed significantly between approaches [*F*(1,22) = 7.377, *p* = 0.013; Table 2]. A *post hoc* pairwise comparison using the Bonferroni correction revealed an overall significantly larger percent of activated voxels for the automated as compared to the manual approach to ROI analysis (automated = 13.23 ± 1.75, manual = 9.945 ± 1.93; *p* = 0.013). Additional within-subjects test results are provided in Table 2. Other main effects and interactions related to the methods comparison were not significant, including interactions between method and group (*p* = 0.546), method and hemisphere (*p* = 0.831), and method, group, and hemisphere (*p* = 0.839).

**Table 2:** **Percent of activated voxels differs between manual and automated approaches to ROI analysis**.

Source	*F* ratio	*p*
**Method**	**7.377**	**0.013**
Method × group	0.373	0.547
**Task**	**35.697**	**0.000**
Task × group	0.030	0.865
Hemisphere	0.310	0.583
Hemisphere × group	0.692	0.414
**Method × task**	**4.838**	**0.039**
Method × task × group	0.005	0.945
Method × hemisphere	0.047	0.831
Method × hemisphere × group	0.042	0.839
Task × hemisphere	0.035	0.853
Task × hemisphere × group	0.207	0.653
Method × task × hemisphere	0.009	0.924
Method × task × hemisphere × group	0.123	0.730

*Within-subjects test results for the main effects of and interactions between method (automated/manual), condition (right hand/left hand action observation), and hemisphere (right/left) on the percent of activated voxels between groups (stroke/control). Significant effects are indicated in bold (*p* < 0.05)*.

## Discussion

These findings demonstrate significant differences between a manual approach and a commonly used automated approach to ROI analysis. The automated approach led to a larger estimated task-related effect size and percent activated voxels compared to the manual approach, in both lesioned and control brains, and in both right and left hemispheres (for participants with stroke, the left hemisphere was the lesioned hemisphere). These findings were consistent across two conditions of cognitive task employed in the fMRI study (5). As discussed, these differences in ROI measures between manual and automated approaches may be attributed to differences in ROI volume, spatial normalization error, and/or spatial smoothing.

Region of interest volume for the left BA 44 was significantly smaller using manual as compared to automated ROI definition, in both groups. ROIs were defined automatically using SPM Anatomy toolbox in which BA 44 is derived from observer-independent analysis of cytoarchitectonic areas and generation of probabilistic maps of 10 post-mortem brains (44), spatially normalized to the T1-weighted MNI structural brain atlas. In Amunts et al., a left greater than right volume asymmetry for BA 44 was identified in all post-mortem brains used to map this brain region (44, 45). Here, no such volume asymmetry was found between the left and right BA 44 using manual ROI definition in lesioned or control brains. The difference between the current findings and those of Amunts et al. is not likely to be age-related, because the mean age in that study was 66 years, and in the current study 65 years for control participants and 66 years for those with stroke. Moreover, in SPM Anatomy toolbox, the left greater than right volume asymmetry survived spatial normalization to the MNI single subject template brain, which was derived from a younger individual (46). The current data do not resolve this discrepancy, but may be more generally representative of inter-individual variability in neuroanatomy and brain volume. A smaller ROI volume may increase effect size by improving statistical power by reducing the number of computed tests, or decrease effect size by capturing fewer activated voxels. Likewise, a larger ROI volume may increase effect size by capturing a larger number of activated voxels, or decrease statistical power by averaging signal over more voxels and increasing the number of statistical tests. Moreover, there was low volume overlap between manual and automated ROIs in both lesioned and control brains, as indicated by low Dice’s coefficients (*d* = 0.2). Several factors may contribute to low volume overlap, including volume differences between manual and automated ROIs, the effects of lesions involving the ROI, experimenter bias utilized in manually defining ROIs, and spatial normalization utilized in automatically defining ROIs. An earlier study reported 70–80% volume overlap (*d* = 0.7–0.8) between manual and automated definitions of the amygdala and hippocampus (47). The hippocampus has clearer anatomical boundaries than the cortical ROIs tested in the current study; that study also did not compare ROI approaches in lesioned brains. An example of the potential effect of ROI volume, in combination with other factors, is provided in Figure 4 from a control participant for whom the larger ROI defined by the automated method captured a larger number of activated voxels from the cluster of interest as compared to the smaller ROI defined by the manual method.

These findings demonstrate significant differences between manual and automated approaches to ROI analysis that are consistent in both lesioned and control brains, indicating that the findings cannot be exclusively attributed to error in spatial normalization of the lesioned brains. The automated method used SPM12’s unified segmentation normalization algorithm, which has been optimized for lesioned brains (16). This approach combines bias correction, tissue segmentation, and spatial normalization in an iterative process to better fit an individual’s brain image to the template brain image (18). Here, there is an overall good fit between individual participant’s brain images and the template brain image (as indicated by a visual check of registration between the images). However, normalization error is always greater in lesioned brains due to intensity changes and/or tissue displacement, and this error is especially problematic when the lesion involves the ROI, as is the case for a number of participants with stroke involving left BA 44. In some cases, experimenter bias (i.e., neuroanatomical expertise) may be necessary to localize an ROI after sulcal changes due to brain injury. An example is provided in Figure 5 for a participant with stroke for whom the experimenter was able to manually define left BA 44, whereas the automated map does not contain the intact tissue from this brain region after tissue displacement due to stroke.

Spatial smoothing may also lead to differences in ROI measures in single subjects. Here, both the automated and manual methods of ROI analysis employed spatial smoothing, in order to improve signal to noise (34). *Post hoc* analysis indicated that the findings hold if the same methods are compared without spatially smoothing the data in either approach [*F*(1,22) = 21.111, *p* = 0.0001], or if smoothing is employed for the automated but not the manual method as is common practice [*F*(1,22) = 14.682, *p* = 0.001]. Spatial smoothing can impact ROI measures if activated voxels fall on the border of an ROI, or if smoothing decreases signal to noise. An example is provided in Figure 6 for a participant with stroke for whom peak task-related activation is localized to the left ventral premotor cortex according to the manual approach (with smoothing), and yet an increase in ROI volume combined with normalizing and smoothing in the automated approach leads to the peak activation being localized to left BA 44.

Despite the significant differences in ROI measures identified in this study between automated and manual approaches, there was a consistent three-way interaction of condition by group by hemisphere using either approach. This finding was reported previously using a similar automated method [(5); SPM8 was used in that study, and ART was not used] and was demonstrated in the current study using both the automated and manual approaches. This consistent group by task interaction across approaches is in line with prior recommendations to utilize task interaction designs in clinical neuroimaging studies, in particular in lesioned brains, rather than testing for main effects between groups (48). By testing for task interactions, any identified group differences cannot be attributed to pathology, such as changes in neurovasculature due to stroke, because those attributes should influence all tasks similarly.

### Limitations

Several limitations of the current study must be considered. First, although BA 44 roughly corresponds to the pars opercularis of the inferior frontal gyrus (44, 45), the cytoarchitectonic areas may not consistently coincide with macroscopic landmarks (e.g., sulci) for this brain region (44), and it is therefore not entirely straightforward to compare them. Nevertheless, BA 44 is often used interchangeably with the designation of pars opercularis of the inferior frontal gyrus in fMRI studies and in those of action observation in particular (49). Thus the current methods comparison is in agreement with commonly used approaches to ROI analysis in such studies. Second, the manual ROIs in this study were drawn by one researcher and the reliability of the drawings was not tested. However, in similar studies, manual ROIs have been drawn by one researcher and compared to automated ROIs [e.g., Ref. (50)], or the accuracy of ROI drawings has been agreed upon by more than one researcher [e.g., Ref. (51)]. Future studies might directly test the inter-rater reliability of manually defined ROIs. Third, the sample size was small, reflecting the difficulty including individuals with chronic stroke and limited mobility in fMRI studies. A similar study used 20 control subjects (47). Statistical power for the methods comparison was also improved by testing two ROIs in each participant (left/right BA 44). Nevertheless, the small sample size limits the generalizability of the findings. The number of ROIs tested was also limited. This study re-analyzed data from an earlier cognitive fMRI study in which activation in BA 44 was hypothesized to be activated by the task and was involved in the lesion in some individuals (5). These attributes made this particular ROI appropriate to use in a comparison of manual and automated approaches to ROI analysis. The number of ROIs involved in the cognitive task and therefore potentially activated and measurable by ROI analysis was limited, as was the number of ROIs both involved in the task and involved in some lesions. Lastly, other preprocessing methods should be considered. Prior studies have used alternative approaches to spatial normalization, such as normalizing with lesion cost function masking (52), which has been shown to reduce errors in a direct comparison of unified segmentation with and without cost function masking (53). However, several automated methods of normalizing brains with lesions have been compared elsewhere, and none appeared to outperform the others (54). Alternative methods of ROI analysis could also be employed, such as using functionally defined ROIs [e.g., Ref. (55)] or alternative probabilistic brain atlases [e.g., Ref. (56)]. The generalizability of the current findings to comparisons between automated and manual approaches to ROI analysis in other ROIs is therefore limited by these and other factors.

## Conclusion

In summary, this study identified significant differences in task-related effect size and percent activated voxels between a manual and automated approach to ROI analysis. These differences were found in lesioned and control brains, in lesioned and non-lesioned hemispheres, and across fMRI task conditions. Therefore it is possible that these findings are generalizable to other ROIs and hypotheses, although further direct testing is warranted. Despite these identified differences, condition by hemisphere by group interactions were consistent across the manual and automated approaches. Although other ROIs and groups should be tested, this finding suggests that a commonly used automated approach to ROI analysis is appropriate for fMRI studies in clinical populations, including individuals with structural brain lesions, although it is recommended that such studies employ a task interaction design. The automated approach is more easily implemented, with minimal requirements for intervention or expertise, thereby minimizing inter-experimenter variability, and is fast and standardized. However, the automated approach is susceptible to spatial normalization error, and the accuracy of ROI definition is only as good as the spatial normalization achieved. Therefore, the automated approach may reduce the validity of ROI comparisons if spatial normalization error decreases sensitivity for the comparison, or if structural differences are incorrectly interpreted as functional differences. In studies with larger cohorts, the problems arising from automated ROI analysis might average out, however, most fMRI studies report statistics in small samples, especially in clinical populations in which recruitment and neuroimaging can prove challenging, such as in individuals with stroke. In these studies, the automated approach tested here may be appropriate if a task interaction design is employed, alternative automated approaches may be tested, or the manual approach should be used. In each, the results of preprocessing should be evaluated at the single subject level and errors corrected or an alternative approach employed.

## Conflict of Interest Statement

The authors declare that the research was conducted in the absence of any commercial or financial relationships that could be construed as a potential conflict of interest.

## Footnotes

^1^http://www.fil.ion.ucl.ac.uk/spm/software/spm12

^2^http://www.nitrc.org/projects/artifact_detect

^3^http://www.mccauslandcenter.sc.edu/mricro/mricron/

^4^http://fsl.fmrib.ox.ac.uk/fsl/fslwiki/Fslutils

^5^http://www.mccauslandcenter.sc.edu/CRNL/tools/spm8-scripts

## References

[1] Jezzard P Buxton RB. The clinical potential of functional magnetic resonance imaging. J Magn Reson Imaging (2006) 23:787–93. 10.1002/jmri.20581 16649209

[2] Ward NS Brown MM Thompson AJ Frackowiak RS. Neural correlates of motor recovery after stroke: a longitudinal fMRI study. Brain (2003) 126:2476–96. 10.1093/brain/awg145 12937084 PMC3717457

[3] Ward NS Brown MM Thompson AJ Frackowiak RS. Neural correlates of outcome after stroke: a cross-sectional fMRI study. Brain (2003) 126:1430–48. 10.1093/brain/awg145 12764063 PMC3717456

[4] Dong Y Winstein CJ Albistegui-Dubois R Dobkin BH. Evolution of fMRI activation in perilesional primary motor cortex and cerebellum with rehabilitation training-related motor gains after stroke: a pilot study. Neurorehabil Neural Repair (2007) 21(5):412–28. 10.1177/1545968306298598 17369516 PMC4067098

[5] Garrison KA Aziz-Zadeh L Wong SW Liew SL Winstein CJ. Modulating the motor system by action observation after stroke. Stroke (2013) 44:2247–53. 10.1161/STROKEAHA.113.001105 23743974 PMC3753677

[6] Stoeckel LE Garrison KA Ghosh S Wighton P Hanlon CA Gilman JM Optimizing real time fMRI neurofeedback for therapeutic discovery and development. Neuroimage Clin (2014) 5:245–55. 10.1016/j.nicl.2014.07.002 25161891 PMC4141981

[7] Friston KJ Worsley KJ Frackowiak RS Mazziotta JC Evans AC. Assessing the significance of focal activations using their spatial extent. Hum Brain Mapp (1994) 1:210–20. 10.1002/hbm.460010306 24578041

[8] Poldrack RA. Region of interest analysis for fMRI. Soc Cogn Affect Neurosci (2007) 2:67–70. 10.1093/scan/nsm006 18985121 PMC2555436

[9] Mitsis GD Iannetti GD Smart TS Tracey I Wise RG. Regions of interest analysis in pharmacological fMRI: how do the definition criteria influence the inferred result? Neuroimage (2008) 40:121–32. 10.1016/j.neuroimage.2007.11.026 18226552

[10] Kimberley TJ Khandekar G Borich M. fMRI reliability in subjects with stroke. Exp Brain Res (2007) 186(1):183–90. 10.1007/s00221-007-1221-8 18060395

[11] Luft AR Forrester L Macko RF McCombe-Waller S Whitall J Villagra F Brain activation of lower extremity movement in chronically impaired stroke survivors. Neuroimage (2005) 26:184–94. 10.1016/j.neuroimage.2005.01.027 15862218

[12] Marshall RS Perera GM Lazar RM Krakauer JW Constatine RC DeLaPaz RL. Evolution of cortical activation during recovery from corticospinal tract infaction. Stroke (2000) 31:656–61. 10.1161/01.STR.31.3.656 10700500

[13] Schnur TT Schwartz MF Kimberg DY Hirshorn E Coslett HB Thompson-Schill SL. Localizing interference during naming: convergent neuroimaging and neuropsychological evidence for the function of Broca’s area. Proc Natl Acad Sci U S A (2009) 106:322–7. 10.1073/pnas.0805874106 19118194 PMC2629229

[14] Solodkin A Hlustik P Noll DC Small SL. Lateralization of motor circuits and handedness during finger movements. Eur J Neurol (2001) 8:425–34. 10.1046/j.1468-1331.2001.00242.x 11554905

[15] Ashburner J Friston K The role of registration and spatial normalization in detecting activations in functional imaging. Clin MRI Dev MR (1997) 7:26–8.

[16] Crinion J Ashburner J Leff A Brett M Price C Friston K. Spatial normalization of lesioned brains: performance evaluation and impact on fMRI analyses. Neuroimage (2007) 37:866–75. 10.1016/j.neuroimage.2007.04.065 17616402 PMC3223520

[17] Price CJ Crinion J Friston KJ. Design and analysis of fMRI studies with neurologically impaired patients. J Magn Reson Imaging (2006) 23:816–26. 10.1002/jmri.20580 16649208

[18] Ashburner J Friston KJ Unified segmentation. Neuroimage (2005) 26:839–51. 10.1016/j.neuroimage.2005.02.018 15955494

[19] Gallese V Fadiga L Fogassi L Rizzolatti G. Action recognition in the premotor cortex. Brain (1996) 119:593–609. 10.1093/brain/119.2.593 8800951

[20] Rizzolatti G The mirror neuron system and its function in humans. Anat Embryol (2005) 210:419–21. 10.1007/s00429-005-0039-z 16222545

[21] Amunts K Weiss PH Mohlberg H Pieperhoff P Eickhoff S Gurd JM Analysis of neural mechanisms underlying verbal fluency in cytoarchitectonically defined stereotaxic space – the roles of Brodmann areas 44 and 45. Neuroimage (2004) 22:42–56. 10.1016/j.neuroimage.2003.12.031 15109996

[22] Rogalsky C Hickok G. The role of Broca’s area in sentence comprehension. J Cogn Neurosci (2011) 23:1664–80. 10.1162/jocn.2010.21530 20617890

[23] Rogalsky C Love T Driscoll D Anderson SW Hickok G. Are mirror neurons the basis of speech perception? Evidence from five cases with damage to the purported human mirror system. Neurocase (2011) 17:178–87. 10.1080/13554794.2010.509318 21207313 PMC3681806

[24] Paulesu E Frith CD Frackowiak RS. The neural correlates of the verbal component of working memory. Nature (1993) 362:342–5. 10.1038/362342a0 8455719

[25] Smith EE Jonides J. Neuroimaging analyses of human working memory. Proc Natl Acad Sci U S A (1998) 95:12061–8. 10.1073/pnas.95.20.12061 9751790 PMC21765

[26] Garrison KA Winstein CJ Aziz-Zadeh L. The mirror neuron system: a neural substrate for methods in stroke rehabilitation. Neurorehabil Neural Repair (2010) 24:404–12. 10.1177/1545968309354536 20207851 PMC11692383

[27] Liew SL KA G Werner J Aziz-Zadeh L The mirror neuron system: innovations and implications for occupational therapy. OTJR Occupation, Participation and Health (2012) 32:79–86. 10.3928/15394492-20111209-01

[28] Hillis AE Gold L Kannan V Cloutman L Kleinman JT Newhart M Site of the ischemic penumbra as a predictor of potential for recovery of functions. Neurology (2008) 71:184–9. 10.1212/01.wnl.0000317091.17339.98 18625964

[29] Jarso S Li M Faria A Davis C Leigh R Sebastian R Distinct mechanisms and timing of language recovery after stroke. Cogn Neuropsychol (2013) 30:454–75. 10.1080/02643294.2013.875467 24472056 PMC3979443

[30] Giussani C Roux FE Ojemann J Sganzerla EP Pirillo D Papagno C. Is preoperative functional magnetic resonance imaging reliable for language areas mapping in brain tumor surgery? Review of language functional magnetic resonance imaging and direct cortical stimulation correlation studies. Neurosurgery (2010) 66:113–20. 10.1227/01.NEU.0000360392.15450.C9 19935438

[31] Oldfield RC The assessment and analysis of handedness: the Edinburgh inventory. Neuropsychologia (1971) 9:97–113. 10.1016/0028-3932(71)90067-4 5146491

[32] Seghier ML Ramlackhansingh A Crinion J Leff AP Price CJ. Lesion identification using unified segmentation-normalisation models and fuzzy clustering. Neuroimage (2008) 41:1253–66. 10.1016/j.neuroimage.2008.03.028 18482850 PMC2724121

[33] Fugl-Meyer AR Jaasko L Leyman I Olsson S Steglind S. The post-stroke hemiplegic patient. 1. a method for evaluation of physical performance. Scand J Rehabil Med (1975) 7:13–31. 1135616

[34] Hopfinger JB Buchel C Holmes AP Friston KJ. A study of analysis parameters that influence the sensitivity of event-related fMRI analyses. Neuroimage (2000) 11:326–33. 10.1006/nimg.2000.0549 10725188

[35] Allen JS Bruss J Brown CK Damasio H. Normal neuroanatomical variation due to age: the major lobes and a parcellation of the temporal region. Neurobiol Aging (2005) 26:1245–60; discussion 1279–1282. 10.1016/j.neurobiolaging.2005.05.023 16046030

[36] Allen JS Damasio H Grabowski TJ. Normal neuroanatomical variation in the human brain: an MRI-volumetric study. Am J Phys Anthropol (2002) 118:341–58. 10.1002/ajpa.10092 12124914

[37] Rorden C Brett M. Stereotaxic display of brain lesions. Behav Neurol (2000) 12:191–200. 10.1155/2000/421719 11568431

[38] Damasio H Human Brain Anatomy in Computerized Images. New York, NY: Oxford University Press (2005).

[39] Eickhoff SB Stephan KE Mohlberg H Grefkes C Fink GR Amunts K A new SPM toolbox for combining probabilistic cytoarchitectonic maps and functional imaging data. Neuroimage (2005) 25(4):1325–35. 10.1016/j.neuroimage.2004.12.034 15850749

[40] Mazziotta JC Toga AW Evans A Fox P Lancaster J A probabilistic atlas of the human brain: theory and rationale for its development. The International Consortium for Brain Mapping (ICBM). Neuroimage (1995) 2:89–101. 10.1006/nimg.1995.1012 9343592

[41] Smith SM Jenkinson M Woolrich MW Beckmann CF Behrens TE Johansen-Berg H Advances in functional and structural MR image analysis and implementation as FSL. Neuroimage (2004) 23(Suppl 1):S208–19. 10.1016/j.neuroimage.2004.07.051 15501092

[42] Dice R Measure of the amount of ecological association between species. Ecology (1945) 26:297–302. 10.2307/1932409

[43] Brett M Anton J Valabregue J “Region of Interest Analysis Using an SPM Toolbox”. Abstract presented at the 8th International Conference on Functional Mapping of the Human Brain, Sendai, Japan, June 2–6 (2002).

[44] Amunts K Schleicher A Burgel U Mohlberg H Uylings HB Zilles K. Broca’s region revisited: cytoarchitecture and intersubject variability. J Comp Neurol (1999) 412:319–41. 10.1002/(SICI)1096-9861(19990920)412:2<319::AID-CNE10>3.0.CO;2-7 10441759

[45] Uylings HB Jacobsen AM Zilles K Amunts K. Left-right asymmetry in volume and number of neurons in adult Broca’s area. Cortex (2006) 42:652–8. 10.1016/S0010-9452(08)70401-5 16881273

[46] Collins DL Zijdenbos AP Kollokian V Sled JG Kabani NJ Holmes CJ Design and construction of a realistic digital brain phantom. IEEE Trans Med Imaging (1998) 17:463–8. 10.1109/42.712135 9735909

[47] Morey RA Petty CM Xu Y Hayes JP Wagner HR II Lewis DV A comparison of automated segmentation and manual tracing for quantifying hippocampal and amygdala volumes. Neuroimage (2009) 45:855–66. 10.1016/j.neuroimage.2008.12.033 19162198 PMC2714773

[48] D’Esposito M Deouell LY Gazzaley A Alterations in the BOLD fMRI signal with ageing and disease: a challenge for neuroimaging. Nat Rev Neurosci (2003) 4:863–72. 10.1038/nrn1246 14595398

[49] Caspers S Zilles K Laird AR Eickhoff SB. ALE meta-analysis of action observation and imitation in the human brain. Neuroimage (2010) 50:1148–67. 10.1016/j.neuroimage.2009.12.112 20056149 PMC4981639

[50] Prodoehl J Yu H Little DM Abraham I Vaillancourt DE. Region of interest template for the human basal ganglia: comparing EPI and standardized space approaches. Neuroimage (2008) 39:956–65. 10.1016/j.neuroimage.2007.09.027 17988895 PMC2253186

[51] Kimberley TJ Birkholz DD Hancock RA VonBank SM Werth TN. Reliability of fMRI during a continuous motor task: assessment of analysis techniques. J Neuroimaging (2008) 18:18–27. 10.1111/j.1552-6569.2007.00163.x 18190491

[52] Brett M Leff AP Rorden C Ashburner J. Spatial normalization of brain images with focal lesions using cost function masking. Neuroimage (2001) 14:486–500. 10.1006/nimg.2001.0845 11467921

[53] Andersen SM Rapcsak SZ Beeson PM. Cost function masking during normalization of brains with focal lesions: still a necessity? Neuroimage (2010) 53:78–84. 10.1016/j.neuroimage.2010.06.003 20542122 PMC2938189

[54] Ripolles P Marco-Pallares J de Diego-Balaguer R Miro J Falip M Juncadella M Analysis of automated methods for spatial normalization of lesioned brains. Neuroimage (2012) 60:1296–306. 10.1016/j.neuroimage.2012.01.094 22305954

[55] Aziz-Zadeh L Wilson SM Rizzolatti G Iacoboni M. Congruent embodied representations for visually presented actions and linguistic phrases describing actions. Curr Biol (2006) 16:1818–23. 10.1016/j.cub.2006.07.060 16979559

[56] Hammers A Allom R Koepp MJ Free SL Myers R Lemieux L Three-dimensional maximum probability atlas of the human brain, with particular reference to the temporal lobe. Hum Brain Mapp (2003) 19:224–47. 10.1002/hbm.10123 12874777 PMC6871794

